# Impact of severity and age with variable definitions of bronchopulmonary dysplasia on neurodevelopmental outcomes

**DOI:** 10.1038/s41390-024-03304-2

**Published:** 2024-06-03

**Authors:** Jack Donlon, Vishwanath Bhat, Krystal Hunter, Alla Kushnir, Vineet Bhandari

**Affiliations:** 1 Cooper Medical School of Rowan University, Camden, NJ USA; 2 Division of Neonatology, Department of Pediatrics, The Children’s Regional Hospital at Cooper, Camden, NJ USA; 3 Cooper Research Institute, Cooper University Hospital, Camden, NJ USA

## Abstract

**Background:**

Bronchopulmonary dysplasia (BPD) is associated with neurodevelopmental impairment (NDI).

**Methods:**

To compare the ability of NICHD 2001 and Jensen 2019 definitions of BPD and respiratory support at 40 weeks postmenstrual age (PMA) to predict NDI, a retrospective study (1/2010–12/2020) was conducted in infants with gestational age <32 weeks and birth weight <1500 g. The primary outcome measure was NDI at 18–24 months corrected age.

**Results:**

Of 1119 infants, 227 (20.7%) met the inclusion criteria and had adequate follow-up data. Multivariate regression analysis showed that the NICHD 2001 definition was not predictive of NDI. Infants with Grade 2 or 3 BPD (Jensen 2019) had 4.75 (95% CI: 1.282–17.563) times greater odds of having NDI than infants without BPD. Infants requiring respiratory support at 40 weeks PMA had 4.95 (95% CI: 1.490–16.47) times greater odds of having NDI. Receiver operating characteristic curves demonstrated that the 2 definitions of BPD and the need for respiratory support at 40 weeks PMA were similar in their ability to predict NDI.

**Conclusion:**

There is no significant difference in the ability of the NICHD 2001 and Jensen 2019 BPD definitions, as well as the need for respiratory support at 40 weeks, for predicting NDI.

**Impact Statement:**

- Current bronchopulmonary dysplasia (BPD) definitions may not effectively predict neurodevelopmental impairment (NDI) in preterm infants.
- Grades 2/3 BPD (Jensen 2019 criteria) significantly associate with NDI.
- Infants requiring respiratory support at 40 weeks post-menstrual age (PMA) have 5 times higher odds of NDI than those on room air at 40 weeks PMA.
- The NICHD 2001, Jensen 2019 definitions, and the requirement for respiratory support at 40 weeks PMA, do not differ in their ability to predict NDI.
- Future studies should include multiple centers, with level III-IV NICUs, catering to socioeconomic, culturally, and racially diverse populations.

## Introduction

Bronchopulmonary dysplasia (BPD) is the most common complication of prematurity,^1^ affecting up to 45% of infants born under 29 weeks of gestational age (GA).^2^ BPD was a highly lethal emphysematous and fibrotic condition when first described over fifty years ago.^3^ However, the characteristics of the condition have changed. Many infants now survive as a result of antenatal corticosteroids use, advanced neonatal care techniques, advancement in respiratory support devices, and surfactant treatment.^4^ BPD is currently described as a clinical syndrome of abnormal lung development, injury, and repair that disrupts alveolarization and microvascular development resulting in abnormal lung mechanics and gas exchange.^5^ Lung development is incomplete in preterm infants and can be further disrupted by standard care provided in the neonatal intensive care unit (NICU) such as supplemental oxygen^6^ and invasive mechanical ventilation.^5,7^ Although BPD is a primary disease of the lungs, it has systemic effects with lifelong consequences and substantial healthcare costs.^8–10^ The clinical presentation and outcomes are heterogeneous, making accurate diagnosis essential to inform the use of respiratory support and medications.^11–13^ Increasing severity of BPD has been associated with an increased prevalence of growth failure, reduced pulmonary function, and neurodevelopmental impairment (NDI).^14–16^ The definition of BPD has undergone multiple revisions,^17–19^ but there is still no definition that accurately predicts future mortality and morbidity.^20^ BPD is one of the few diseases where the diagnostic criteria are based on the treatment given, rather than on pathophysiology, clinical picture, or diagnostic tests.^21^ The definitions rely on the need for oxygen or respiratory support, which are often subjective and vary across medical centers.^22^ The current definitions do not adequately define the severity of the disease, and it is inconclusive if they can reliably predict long-term neurodevelopmental outcomes.^23^ There have been few studies published to date on the impact of severity and timing of diagnosis of BPD on neurodevelopmental outcomes.^15,16,24–27^ The current studies are limited, and have shown mixed results. However, these results suggest that infants with severe BPD according to the National Institute of Child Health and Human Development^17^ (NICHD 2001) have significantly higher odds of developing NDI compared to infants without BPD.^15^ Infants with Grade 3 BPD according to the Jensen et al. ^19^, (Jensen 2019) definition of BPD have also been found to have significantly higher odds of developing NDI compared to infants without BPD.^16^ Additionally, infants requiring respiratory support at 40 weeks postmenstrual age (PMA) compared to infants who did not require respiratory support at 40 weeks PMA have been found to have higher odds of developing NDI.^15^

The first objective of this study was to compare the abilities of the NICHD 2001^17^ and the Jensen 2019^19^ definitions of BPD to predict neurodevelopmental outcomes at a corrected age (CA) of 18–24 months. The second objective was to determine if the requirement of respiratory support at 40 weeks PMA is a better predictor of NDI at 18–24 months CA compared to the requirement of respiratory support at 36 weeks PMA.

## Methods

### Study subjects

This was a retrospective cohort study conducted at The Children’s Regional Hospital at Cooper/Cooper University Hospital (CUH) in Camden, NJ. CUH has a Level-III NICU and an outpatient, neonatal follow-up clinic. The study was approved by the CUH research ethics board (IRB-22-095). Infants included in this study were born between January 2010 and December 2020. They were identified from the CUH neonatal database (Neonatal Information System). Infants born under 32 weeks GA with a birth weight (BW) under 1500 g were eligible to be included in the study. Exclusion criteria were the presence of congenital or genetic anomalies, death before discharge, or transfer to another center resulting in insufficient documentation.

### Data collection

Data was collected using NIS and hospital electronic medical records system (EPIC). The first author (JD) reviewed admission notes, daily progress notes, and discharge summaries from infants’ electronic medical records to collect demographic, delivery, NICU course, and respiratory support data. Respiratory data was reviewed to retrospectively to classify infants with different severity or grade of BPD following the NICHD 2001^17^ and Jensen 2019^19^ criteria. Patients were classified based on respiratory support at 28 days of life and then further evaluated at 36 weeks PMA. Respiratory support at 40 weeks PMA was defined as the need for any intervention at 40 weeks. This included infants who required nasal canula, continuous positive airway pressure, nasal intermittent positive pressure ventilation, and mechanical ventilation. The 2nd and senior authors (VB and VB) confirmed the data and classifications.

Physician and developmental psychologists’ follow-up notes at 18–24 months CA were reviewed by the first author (JD) to collect outcome data. Outcome data wwere reviewed to differentiate between infants with and without NDI. NDI was defined as blindness, deafness, or a composite score of less than 85 on the cognitive, language, or motor components of the third edition of the Bayley Scale of Infant and Toddler Development (BSID-III).^28–30^ Hearing impairment was defined as absence of useful hearing even with aids, and vision impairment was defined as bilateral visual acuity <20/200. We did not use Gross Motor Function Classification Score for diagnosis of cerebral palsy. BSID-III evaluations were completed by a single developmental psychologist who was blinded to the BPD classifications in terms of NIH 2001 and Jensen 2019 definitions, as these were done independently. Children with significant disabilities impeding completion of developmental testing were included in the NDI group. NDI classifications were confirmed by the 2^nd^ author (VB) who was blinded to the BPD classifications.

### Statistics

Chi-squared or Fisher’s exact test compared categorical variables between infants with and without BPD. Independent t-tests and Mann-Whitney U tests compared continuous variables between infants with and without BPD. Multivariate logistic regression analysis models evaluated the association between the two definitions of BPD (NICHD 2001 and Jensen 2019) and NDI. Infants without BPD served as the control group for these two models. An additional model evaluated the association between the need for respiratory support at 40 weeks PMA with NDI. Infants who did not require respiratory support at 40 weeks served as the control group for this model. This includes any infant who did not require a respiratory intervention at 40 weeks PMA. Variables known to be predictors of poor neurodevelopment were included in data collection, to allow for adjustment of confounders within the multivariate logistic regression analysis models.^15,16,24,31^ The models adjusted for confounders that were unequally distributed among the groups (Tables 1–3).

**Table 1:** Comparison of maternal and neonatal characteristics of infants with different severity of BPD—NIH 2001 definition (*N* = 227).

	No BPD (*N* = 75)	NIH 2001—Mild (*N* = 67)	NIH 2001—Moderate (*N* = 56)	NIH 2001—Severe (*N* = 29)	*P* value
*Maternal characteristics*					
Antenatal Steroids (≥2 doses)	69 (92.0%)	62 (92.5%)	53 (94.6%)	25 (86.2%)	0.595
Cesarean Section	39 (52.0%)	26 (38.8%)	35 (62.5%)	18 (62.1%)	**0.040**
Chorioamnionitis	7 (9.3%)	9 (13.4%)	8 (14.3%)	1 (3.4%)	0.404
Maternal Diabetes	10 (13.3%)	9 (13.4%)	8 (14.5%)	4 (13.8%)	0.997
PIH or Pre-eclampsia	40 (53.3%)	16 (23.9%)	19 (33.9%)	10 (34.5%)	**0.003**
*Neonatal characteristics*					
Birth Weight (grams) (±SD)	1181 (205.22)	959.91 (205.37)	919.59 (195.08)	710.28 (207.79)	**<0.001**
Gestational Age (weeks) (±SD)	30.10 (1.27)	27.30 (1.73)	27.34 (2.01)	25.78 (1.83)	**<0.001**
Race					
White	26 (34.7%)	16 (23.9%)	19 (33.9%)	6 (20.7%)	
Black	32 (42.7%)	28 (41.8%)	18 (32.1%)	17 (58.6%)	0.121
Hispanic	11 (14.7%)	19 (28.4%)	17 (30.4%)	3 (10.3%)	
Other	6 (8.0%)	4 (6.0%)	2 (3.6%)	3 (10.3%)	
Male Sex	32 (42.7%)	37 (55.2%)	33 (58.9%)	16 (55.2%)	0.252
SGA	14 (18.7%)	6 (9.0%)	7 (13.0%)	5 (17.2%)	0.388
Delivery Room Intubation	21 (26.9%)	40 (59.7%)	37 (66.1%)	27 (93.1%)	**<0.001**
Surfactant use	19 (24.4%)	43 (64.2%)	43 (76.8%)	29 (100.0%)	**<0.001**
Days of Mechanical Ventilation (IQR)	0 (0–1)	2 (0-6)	8 (2-26)	40 (17-51.5)	**<0.001**
PDA requiring treatment	0 (0.0%)	11 (16.4%)	15 (26.8%)	12 (41.4%)	**<0.001**
IVH ≥ Grade 3	4 (5.3%)	5 (7.5%)	4 (7.1%)	5 (17.2%)	0.396
Culture Positive Sepsis	34 (45.3%)	36 (53.7%)	37 (66.1%)	21 (72.4%)	**0.028**
NEC ≥ Stage 2	1 (1.3%)	3 (4.5%)	2 (3.6%)	5 (17.2%)	**0.008**
Cystic PVL	6 (8.0%)	5 (7.1%)	4 (7.0%)	3 (10.0%)	0.962
ROP requiring treatment	0 (0.0%)	1 (1.5%)	7 (12.5%)	7 (24.1%)	**<0.001**
Postnatal Steroids	2 (2.7%)	7 (10.4%)	18 (32.1%)	21 (72.4%)	**<0.001**

The *p* value listed for each variable compared no BPD to each specific severity of BPD.

*BPD* bronchopulmonary dysplasia, *IQR* interquartile range, *IVH* intraventricular hemorrhage, *NEC* necrotizing enterocolitis, *PDA* patent ductus arteriosus, *PIH* pregnancy-induced hypertension, *PVL* periventricular leukomalacia, *ROP* retinopathy of prematurity, *SD* standard deviation, *SGA* small for gestational age.

**Table 2:** Comparison of maternal and neonatal characteristics of infants with different severity of BPD—Jensen 2019 definition (*N* = 227).

	Jensen 2019—No BPD (*N* = 141)	Jensen 2019—Grade 1 BPD (*N* = 63)	Jensen 2019—Grade 2/3 BPD (*N* = 23)	*P* value
*Maternal characteristics*				
Antenatal Steroids (≥2 doses)	130 (92.2%)	59 (93.7%)	20 (87.0%)	0.594
Cesarean Section	64 (45.4%)	39 (61.9%)	15 (65.2%)	**0.038**
Chorioamnionitis	16 (11.3%)	9 (14.3%)	0 (0.0%)	0.169
Maternal Diabetes	19 (13.5%)	8 (12.9%)	4 (17.4%)	0.859
PIH or Pre-eclampsia	56 (39.7%)	23 (36.5%)	6 (26.1%)	0.449
*Neonatal Characteristics*				
Birth Weight (grams) (±SD)	1079.40 (231.28)	885.21 (219.94)	728.96 (193.38)	**<0.001**
Gestational Age (weeks) (±SD)	28.81 (2.03)	27.13 (2.11)	25.81 (1.67)	**<0.001**
Race				
White	41 (29.1%)	23 (36.5%)	3 (12.5%)	
Black	60 (42.6%)	22 (34.9%)	14 (58.3%)	0.218
Hispanic	30 (21.3%)	16 (25.4%)	4 (16.7%)	
Other	10 (7.1%)	2 (3.2%)	3 (13.0%)	
Male Sex	69 (48.9%)	34 (54.0%)	15 (65.2%)	0.327
SGA	20 (14.2%)	9 (14.8%)	3 (13.0%)	0.980
Delivery Room Intubation	60 (42.6%)	43 (68.3%)	22 (95.7%)	**<0.001**
Surfactant use	61 (43.3%)	50 (79.4%)	23 (100%)	**<0.001**
Days of Mechanical Ventilation (IQR)	0 (0-2)	9 (2-28.5)	42 (16-52)	**<0.001**
PDA requiring treatment	10 (7.1%)	17 (27.0%)	11 (47.8%)	**<0.001**
IVH ≥ Grade 3	11 (7.8%)	5 (7.9%)	4 (17.4%)	0.309
Culture Positive Sepsis	69 (48.9%)	42 (66.7%)	17 (73.9%)	**0.013**
NEC ≥ Stage 2	4 (2.8%)	2 (3.2%)	5 (21.7%)	**≤0.001**
Cystic PVL	11 (7.8%)	4 (6.3%)	3 (12.5%)	0.612
ROP requiring treatment	0 (0.0%)	9 (14.3%)	6 (26.1%)	**<0.001**
Postnatal Steroids	9 (6.4%)	23 (36.5%)	16 (69.6%)	**<0.001**

The *p* value listed for each variable compared no BPD to each specific severity of BPD.

*BPD* bronchopulmonary dysplasia, *IQR* interquartile range, *IVH* intraventricular hemorrhage, *NEC* necrotizing enterocolitis, *PDA* patent ductus arteriosus, *PIH* pregnancy-induced hypertension, *PVL* periventricular leukomalacia, *ROP* retinopathy of prematurity, *SD* standard deviation, *SGA* small for gestational age.

**Table 3:** Comparison of maternal and neonatal characteristics of infants with and without respiratory support at 40 weeks gestational age (*N* = 227).

	No respiratory support at 40 weeks gestational age (*N* = 196)	Respiratory support at 40 weeks gestational age (*N* = 31)	*P* value
*Maternal characteristics*			
Antenatal Steroids (≥2 doses)	182 (92.9%)	27 (87.1%)	0.281
Cesarean Section	96 (49.0%)	22 (71.0%)	**0.023**
Chorioamnionitis	23 (11.7%)	2 (6.5%)	0.543
Maternal Diabetes	26 (13.3%)	5 (16.1%)	0.778
PIH or Pre-eclampsia	75 (38.3%)	10 (32.3%)	0.521
*Neonatal Characteristics*			
Birth Weight (grams) (±SD)	1038.45 (233.09)	683.61 (156.21)	**<0.001**
Gestational Age (weeks) (±SD)	28.43 (2.11)	25.54 (1.48)	**<0.001**
Race			
White	63 (32.1%)	4 (12.9%)	
Black	78 (39.8%)	17 (54.8%)	0.169
Hispanic	42 (21.4%)	8 (25.8%)	
Other	13 (6.6%)	2 (6.5%)	
Male Sex	103 (52.6%)	15 (48.4%)	0.666
SGA	26 (13.3%)	6 (19.4%)	0.446
Delivery Room Intubation	95 (48.5%)	30 (96.8%)	**<0.001**
Surfactant use	105 (53.6%)	29 (93.5%)	**<0.001**
Days of Mechanical Ventilation (IQR)	1 (0-6)	37 (18.75-49.25)	**<0.001**
PDA requiring treatment	24 (12.2%)	14 (44.2%)	**<0.001**
IVH ≥ Grade 3	13 (6.6%)	7 (22.6%)	**0.004**
Culture Positive Sepsis	104 (53.1%)	24 (77.4%)	**0.011**
NEC ≥ Stage 2	9 (4.6%)	2 (6.5%)	0.650
Cystic PVL	14 (7.1%)	4 (12.9%)	0.281
ROP requiring treatment	6 (3.1%)	9 (29.0%)	**<0.001**
Postnatal Steroids	25 (12.8%)	23 (74.2%)	**<0.001**

*IQR* Interquartile Range, *IVH* intraventricular hemorrhage, *NEC* necrotizing enterocolitis, *PDA* patent ductus arteriosus, *PIH* pregnancy-induced hypertension, *PVL* Periventricular leukomalacia, *ROP* retinopathy of prematurity, *SD* standard deviation, *SGA* small for gestational age.

*Indicates *p*  < 0.05.

Associations are provided as odds ratios (OR) with 95% confidence intervals (CI). Probability values (*p* value) below 0.05 were considered significant, and testing was two-sided. Odds ratios and their corresponding confidence intervals were directly compared to compare the predictive abilities of both definitions of BPD and the requirement of respiratory support at 40 weeks PMA. Receiver operating characteristic (ROC) curves were generated to quantify the area under the curve (AUC) for each model. Additionally, the predictive values (sensitivity, specificity, positive predictive value (PPV), negative predictive value (NPV), and accuracy) for NDI of each severity of BPD were calculated using the group of infants without BPD as a reference. The predictive values for the need for respiratory support at 40 weeks PMA were also calculated. All statistical analyses were performed using IBM SPSS Statistics for Windows, version 27.

## Results

### Study subjects

**Fig. 1:**
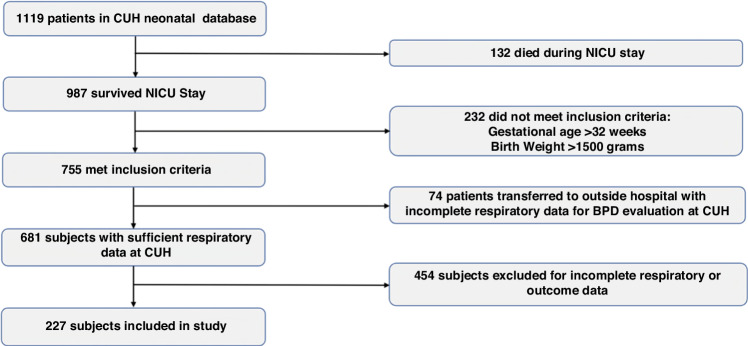
A total of 1119 infants born between January 2010 and December 2020 were included in the CUH neonatal database. Of the 1119 infants, 132 (11.8%) died in the NICU and were excluded. Of the remaining 987 subjects, 232 (20.7%) did not meet the gestational age or birthweight inclusion criteria. An additional 74 (6.6%) of the remaining 755 subjects were excluded because they were transferred to an outside hospital and did not have sufficient data. Another 453 (40.5%) of the remaining 681 subjects were excluded from the study due to a lack of follow-up data. One additional subject was removed for having insufficient respiratory data. The study included 227 subjects (20.3%). BPD bronchopulmonary dysplasia, CUH Cooper University Hospital, NICU neonatal intensive care unit.

The CUH neonatal database included 1119 infants born between January 2010 and December 2020. Of the 1119 infants, 132 (11.8%) died in the NICU and were excluded from the study. Of the remaining 987 subjects, 232 (20.7%) did not meet the GA or BW inclusion criteria. An additional 74 (6.6%) of the remaining 755 subjects were excluded because they were transferred to an outside hospital and did not have sufficient data. Another 453 (40.5%) of the remaining 681 subjects were excluded from the study because NDI was not assessed by the developmental psychologist. One additional subject was excluded for having insufficient respiratory data. This left 227 subjects to be included in the study (20.3%). Figure 1 displays the study flow chart.

### Bronchopulmonary dysplasia prevalence

The respiratory data of the 227 subjects were analyzed to retrospectively categorize subjects into the NICHD 2001 and the Jensen 2019 definitions of BPD. A total of 152 (66.9%) had BPD, and 75 did not have BPD (33.0%) following the NICHD 2001 definition. Among the 152 subjects with BPD, 67 (44.1%) were mild, 56 (36.8%) were moderate, and 29 (19.1%) were severe. A total of 86 of the 227 subjects (37.9%) had BPD, and 141 (62.1%) did not have BPD following the Jensen 2019 definition. Among the 86 subjects, 63 (73.3%) were categorized as Grade 1, 19 (22.1%) were Grade 2, and 4 (4.7%) were Grade 3. Additionally, 31 (13.7%) of the 227 subjects required oxygen supplementation or respiratory support at 40 weeks PMA.

### Covariates

The following variables (Tables 1–3) were significantly different in infants without BPD and with different severities of BPD and among subjects not requiring and requiring respiratory support at 40 weeks: BW, cesarean section, culture-positive sepsis, days of mechanical ventilation, delivery room intubation, GA, patent ductus arteriosus (PDA) requiring treatment, postnatal steroid administration, retinopathy of prematurity (ROP) requiring treatment, and surfactant use. BW and GA were significantly lower in infants with any severity of BPD in both definitions as compared to infants without BPD. BW and GA were also lower when comparing infants who did require respiratory support at 40 weeks PMA to infants who did not require respiratory support at 40 weeks PMA. The other variables listed were more prevalent or had higher values in infants with any severity of BPD in both definitions as compared to infants without BPD. The other variables listed were also more prevalent or had higher values in infants who required respiratory support at 40 weeks PMA. The prevalence of necrotizing enterocolitis (NEC) was significantly different in infants without BPD and with the different severities of the NICHD 2001 and Jensen 2019 definitions of BPD. The prevalence of NEC was not significantly different in infants who required respiratory support at 40 weeks as compared to those who did not. The prevalence of pregnancy-induced hypertension or pre-eclampsia was significantly different in infants without BPD and with the different severities of the NICHD 2001 definition of BPD. The prevalence of intraventricular hemorrhage (IVH) was different in infants who required respiratory support at 40 weeks PMA compared to those who did not.

BW, culture-positive sepsis, GA, IVH, NEC, PDA requiring treatment, postnatal steroid administration, and PIH were adjusted for in the multivariate logistic regression analysis models as they were unequally distributed among the groups (Tables 1–3). These variables are also known predictors of poor neurodevelopment and have been adjusted for in similar studies.^13,15,16,24,31^ Days of mechanical ventilation, delivery room intubation, and surfactant use were not adjusted. The definition and development of BPD is dependent upon these variables.

### Neurodevelopmental outcomes

At 18–24 months CA, 84 (37.0%) of 227 subjects had NDI. Three multivariate logistic regression analysis models were generated to determine associations between the NICHD 2001 definition of BPD, the Jensen 2019 definition of BPD, and the need for respiratory support at 40 weeks PMA with NDI (Table 4). The models adjusted for potential confounding variables. There was no statistically significant difference in the prevalence of NDI in subjects with mild, moderate, or severe BPD as compared to infants without BPD. There was also no statistically significant difference in the prevalence of NDI in subjects with Grade 1 or 2 BPD compared to infants without BPD. Grade 3 BPD could not be evaluated separately in the multivariate logistic regression analysis model because only four subjects met the criteria. Each of these subjects developed NDI.

**Table 4:** Multivariate logistic regression analysis.

Outcome = NDI	Odds Ratio	95% confidence interval	
		Lower	Upper
NICHD 2001^17^ definition of BPD			
No BPD vs. Mild	0.647	0.242	1.728
No BPD vs. Moderate	0.871	0.313	2.420
No BPD vs. Severe	3.618	0.906	14.451
Jensen 2019^19^ definition of BPD			
No BPD vs. Grade 1	1.220	0.551	2.702
No BPD vs. Grade 2	3.179	0.847	11.927
No BPD vs Grade 2/3	**4.745**	**1.282**	**17.563**
Respiratory support at 40 weeks PMA			
40 weeks PMA	**4.953**	**1.490**	**16.47**

NDI was assessed at 18–24 months corrected age. NDI was defined as blindness, deafness, or a composite score of less than 85 on the cognitive, language, or motor components of the third edition of the Bayley Scale of Infant and Toddler Development (BSID-III).^28^ The multivariate logistic regression analysis model could not support Grade 3 BPD because of the sample size. Only four subjects met the criteria for Grade 3 BPD. All subjects with Grade 3 BPD developed NDI. Grade 2/3 BPD results were determined by including all the infants with Grade 2 and Grade 3 BPD in the same group in the model.

*BPD* bronchopulmonary dysplasia, *NDI* neurodevelopmental impairment, *NICHD* National Institute of Child Health and Human Development, *PMA* postmenstrual age.

The multivariate logistic regression analysis model was reevaluated with Grade 2 and Grade 3 BPD combined into a single group (Grade 2/3 BPD). Subjects in the combined Grade 2/3 BPD group had 4.75 times greater odds of developing NDI (95% CI: 1.282–17.563) (Table 4). Subjects on respiratory support at 40 weeks had 4.95 times greater odds to have NDI (95% CI: 1.490–16.47).

ROC curves were generated to compare the predictive abilities of each BPD definition for NDI. The model generated for the NICHD 2001 definition had an AUC of 0.778 (95% CI: 0.714–0.842). The AUC for the Jensen 2019 definition was 0.765 (95% CI: 0.700–0.830). The AUC for respiratory support at 40 weeks PMA was 0.770 (95% CI: 0.707–0.835).

In general, both the NICHD 2001 and the Jensen 2019 definitions of BPD as well as the need for respiratory support at 40 weeks PMA had poor sensitivity while Grades 2 or 3 BPD by Jensen 2019 definition and respiratory support at 40 weeks PMA had high (94%) specificity for predicting NDI at 18–24 months CA (Table 5).

**Table 5:** Predictive values of BPD for NDI.

	NICHD 2001^17^			Jensen 2019^19^			Respiratory support at 40 weeks
	Mild	Moderate	Severe	Grade 1	Grade 2	Grade 3	
Sensitivity	0.477	0.452	0.477	0.343	0.279	0.262	0.262
Specificity	0.531	0.584	0.867	0.708	0.942	0.937	0.937
PPV	0.313	0.339	0.724	0.365	0.739	0.710	0.710
NPV	0.693	0.693	0.693	0.688	0.688	0.684	0.684
Accuracy	0.514	0.542	0.702	0.588	0.695	0.687	0.687

The predictive values of the NICHD 2001 and Jensen 2019 definitions of BPD for NDI were calculated using infants without BPD as a reference. The entire cohort was included in the predictive testing of respiratory support at 40 weeks for NDI. NDI was assessed at 18–24 months of corrected age. NDI was defined as blindness, deafness, or a composite score of less than 85 on the cognitive, language, or motor components of the third edition of the Bayley Scale of Infant and Toddler Development (BSID-III).^28^

*BPD* bronchopulmonary dysplasia, *NDI* neurodevelopmental impairment, *NICHD* National Institute of ChildHealth and Human Development.

## Discussion

### Key findings

In this study, approximately two-thirds of infants met the NICHD 2001 criteria for a diagnosis of BPD, and just over one-third met the Jensen 2019 criteria. NDI was identified in slightly more than one-third of the subject population. The mild, moderate, and severe classifications from the NICHD 2001 definition of BPD did not exhibit statistical significance in predicting NDI. Additionally, the Grade 1 and 2 BPD classifications from the Jensen 2019 definition of BPD did not exhibit statistical significance in predicting NDI. All 4 infants that met the criteria for Grade 3 BPD developed NDI. Therefore, it was not possible to include Grade 3 BPD in the multivariate logistic regression model. The multivariate logistic regression analysis model was reevaluated with Grade 2 and Grade 3 BPD combined into a single group (Grade 2/3 BPD). Grades 2/3 BPD significantly associated with NDI. Furthermore, infants requiring respiratory support at 40 weeks PMA had almost five times greater odds to have NDI than infants who did not require respiratory support at 40 weeks PMA. A direct comparison of the odds ratios and confidence intervals for the two definitions of BPD and the requirement of respiratory support at 40 weeks PMA indicated no significant difference in their predictive abilities. ROC curves further demonstrated that the NICHD 2001 and Jensen 2019 definitions of BPD, along with the need for respiratory support at 40 weeks PMA, had similar predictive abilities for NDI at 18–24 months CA.

### Comparison to the existing literature

The average BW and GA of this population were comparable to the populations studied by Malavolti et al., Han et al., and Katz et al. ^15,16,27^ The prevalence of BPD according to the NICHD 2001 definition (66.9%) was comparable to 58.5% identified by Malavolti et al., and 66.0% identified by Han et al., but not the 38.9% identified by Katz et al. ^15,16,27^ The prevalence of BPD according to the Jensen 2019 definition (37.9%) was comparable to the 30% identified by Han et al. ^16^ Jeon et al., studied a population with lower average GA and BW.^24^ Prevalence of BPD was higher in the population included in their study.^24^ This is consistent with the evidence that GA and BW have an inverse relationship with BPD.^13^ The variables that we identified that were significantly different among different BPD severities (Tables 1–3**)** were consistent with similar studies in the literature.^13,15,16,24,32^

The prevalence of NDI in this population (37%) was higher than the prevalence of NDI seen in Malavolti et al. (16.1%), Han et al. (28.2%), Jeon et al. (23.3%), and Katz et al. (18.0%).^15,16,24,27^ This may be the result of a smaller sample size. It may also be a characteristic of the patient population. CUH caters to a resource-limited urban community. The multivariate regression analysis models demonstrated a stepwise increase in the prevalence of NDI across both the NICHD 2001 and Jensen 2019 definitions of BPD. However, mild, moderate, and severe BPD did not predict NDI with statistical significance. This result agrees with the results demonstrated by Han et al., and Jeon et al. However, they disagree with Malavolti et al., who found that severe BPD associated with NDI with significance.^15^ Additionally, Grade 1 or Grade 2 BPD did not predict NDI with statistical significance. These results do not agree with Han et al., who found a statistically significant association between Grade 1 BPD and NDI and Jeon et al., who found a statistically significant association between Grade 2 BPD and NDI.^16,24^ Han et al. also found that Grade 3 BPD significantly associated with NDI.^16^ The multivariate logistic regression model of the Jensen 2019 definition designed for this study could not support Grade 3 BPD because of limited sample size. Of note, all 4 subjects classified with Grade 3 BPD developed NDI. The multivariate regression analysis models also determined that there was a statistically significant association between the requirement of respiratory support at 40 weeks PMA with NDI. This confirms the results found by Malavolti et al. (Table 4).^15^ The Jensen 2019 definition of BPD was more sensitive in predicting NDI as compared to the NICHD 2001 definition of BPD (Table 5). Grade 2 and 3 BPD were 94.2% and 93.7% sensitive for NDI, respectively. The requirement of respiratory support at 40 weeks PMA was also 93.7% sensitive for NDI. These results agree with Han et al. Cocuzzo et al. reported that the negative and positive predictive values for NDI were similar for Jensen 2019 and NIH 2001 definitions.^33^ The Jensen 2019 definition was more sensitive to determine NDI when there was no BPD, while the NICHD 2001 definition was more specific to determine severe NDI when moderate or severe BPD was present.^33^

This is the second study to use ROC curves to compare the predictive abilities of BPD definitions. This study found higher AUC results for the NICHD 2001 (AUC = 0.778) and the Jensen 2019 (0.765) definition compared to Katz et al. (NICHD 2001 AUC = 0.65, Jensen 2019 AUC = 0.63). Katz et al, similarly did not find a significant difference between the NICHD 2001 and Jensen 2019 definitions in predicting NDI at a 2-year corrected age follow-up.

### The leading definitions of bronchopulmonary dysplasia

Each definition of BPD has its limitations. The NICHD 2001 definition of BPD is poorly reflective of current respiratory support strategies^34^ with the “mild” category of BPD having limited predictive value for long-term morbidity.^14^ Moving the time point of assessment to 40 weeks PMA may be difficult because many infants will have already met the criteria for discharge. Only 31 (13.7%) subjects required respiratory support at 40 weeks PMA in this study. Infants breathing room air without respiratory support at 36 weeks PMA are unlikely to require oxygen or respiratory support at 40 weeks PMA.^13^ However, infants may be classified as having BPD at 40 weeks if they are on modest oxygen requirements despite meeting all the other prerequisites for discharge.^13^ The Jensen 2019 criteria were established in a retrospective study that tested 18 potential definitions against outcome data obtained from the NICHD Neonatal Research Network.^19^ This criteria separates infants dependent on invasive versus non-invasive ventilation or nasal cannula with a fraction of inspired oxygen (FiO_2_) > 0.3. This is beneficial when evaluating the impact of novel therapies designed to mitigate the severity of BPD. The criteria are also easy to apply in clinical practice and research studies because of the omission of the oxygen reduction test.^13^ Despite these advantages, it did not prove to be a superior definition in this study. The average GA in the study conducted by Jensen et al. was 25.2 weeks (standard deviation = 1.3 weeks). However, our study and several similar studies included populations with a higher mean GA.^15,16,27^ It is possible that these discrepant findings may be secondary to the difference in the population studied.

The NICHD 2018 criteria were not included within this study as a 2021 study (*N* = 393) found the NICHD criteria to be a better predictor of late death or serious respiratory morbidity.^35^ Katz et al., also found it to have less predictive power for neurologic outcomes compared to the NICHD 2001 and Jensen 2019 definitions.^27^ This was the first BPD definition that accounted for early death (between 14 days and 36 weeks PMA) from respiratory failure.^18^ This study did not measure early death due to respiratory failure as an outcome measure. Several studies have included early death as an outcome measure,^24,25,27,35^ but others have also made this omission.^15,16,26^ Future studies may consider including early death as an outcome measure as it may be relevant in a future definition that emerges as superior.

### Study strengths

The strengths of this study include a racially and ethnically diverse population and consistency in care and follow-up evaluations. CUH is an urban, tertiary, regional center, and the racial breakdown of the study population was 67 (29.5%) white, 50 (22.0%) Hispanic, 95 (41.8%) Black, 9 (3.96%) Asian, and 6 (2.64%) other races. This will allow results to be generalizable to other US inner-city, urban, academic Level IIIB NICUs. To the best of our knowledge, this is the first US study that compares the predictive abilities of all three BPD definitions. Additionally, the study was only carried out at CUH. Therefore, treatment provided to subjects was likely more consistent than in multicenter studies. Oxygen supplementation and respiratory support are often subjective and vary across medical centers.^22^ The BSID-III was the only measure used to evaluate neurodevelopmental outcomes whereas other studies assessed infants with a variety of scoring systems.^15,16,24,26^ There was also no inter-rater bias because all developmental evaluations (BSID-III) were completed by one developmental psychologist at the neonatal outpatient clinic.

### Study limitations and future directions

Limitations of this study include sample size, follow-up, and a 10-year study period. The neonatal database contained 1119 patients whereas Han et al., drew from a database with 8294 patients.^16^ It was not possible to evaluate a group of subjects with Grade 3 BPD because of limited sample size. Only 227 of the 681 (33.3%) subjects who had sufficient respiratory data, and met the inclusion criteria, had follow-up at 18–24 months CA for neurodevelopmental evaluation. The follow-up rate in the present study was lower than similar studies,^15,16,24^ and may be reflective of the patient population at CUH and differences in the US healthcare system. Many infants with less severe BPD and/or minimal or no NDI failed to return for follow up clinic at 18–24 months CA because they were developmentally normal. Additionally, some infants with high-severity BPD and other co-morbidities were transferred to neighboring higher-level care centers, and thus may not have been followed at CUH to evaluate for NDI. However, we believe the loss to follow-up is representative of similar inner-city urban NICUs with a catchment area serving predominantly the underserved. Approximately 80% of our NICU patients are covered by Medicaid. Data was collected from January 2010 to December 2020. This represents an additional limitation as longer periods of study increase the potential for unmeasured confounders and changes in care practices.

MRI may be indicated to visualize the brains of infants who are at high risk for white matter injury (<29 weeks, <1000 g) as it provides more detailed images of these injuries than cranial ultrasound.^36^ Therefore, future studies should consider collecting MRI data if available. Future studies may also consider collecting data on maternal age, socio-economic status (SES), and degree of NDI. Maternal age and SES may act as confounding variables, impacting developmental outcomes. A score <85 in the BSID-III indicates mild impairment whereas a score <70 indicates moderate or severe impairment. Stratifying the severity of impairment was not considered within the scope of the present study. These limitations should be considered by investigators designing similar studies. To remedy challenges with sample size and follow-up, data from multiple centers, serving populations with a range of socio-economic characteristics would be helpful. Inclusion of centers with levels III-IV NICUs may also allow for informative sub-analysis.

## Conclusions

The results of this study demonstrate that the requirement of respiratory support at 40 weeks PMA significantly associates with NDI. There is not a significant difference in the abilities of the NICHD 2001 definition of BPD, the Jensen 2019 definition of BPD, and the need for respiratory support at 40 weeks PMA to predict NDI. Each of these definitions have limitations, and results from similar studies are not entirely consistent. Additional multi-center studies are warranted to compare the predictive abilities of each definition. The definition of BPD will continue to need revision if no definition proves to be superior in predicting consequential outcomes like NDI. Advances in clinical practice and changes in the at-risk population will continue to drive further revision.

## Data Availability

The data analyzed in this study is not publicly available. Datasets are available from the corresponding author upon reasonable request.
