# Correction: Impact of severity and age with variable definitions of bronchopulmonary dysplasia on neurodevelopmental outcomes

**DOI:** 10.1038/s41390-024-03417-8

**Published:** 2024-07-22

**Authors:** Jack Donlon, Vishwanath Bhat, Krystal Hunter, Alla Kushnir, Vineet Bhandari

**Affiliations:** 1 Cooper Medical School of Rowan University, Camden, NJ USA; 2 Division of Neonatology, Department of Pediatrics, The Children’s Regional Hospital at Cooper, Camden, NJ USA; 3 Cooper Research Institute, Cooper University Hospital, Camden, NJ USA

Correction to: *Pediatric Research* 10.1038/s41390-024-03304-2, published online 3 June 2024

In the original version of this article, some mistakes were detected in two references. Reference 32 was corrected from “Sahni, M. Bronchopulmonary Dysplasia. StatPearls Publishing, Treasure Island (FL)” to “Sahni, M. & Mowes, A.K. Bronchopulmonary Dysplasia. StatPearls Publishing, Treasure Island (FL). (2023)”. Reference 33 was corrected from “Cocuzzo. Prediction of Neurodevelopmental Impairment Based on Severity of Bronchopulmonary Dysplasia as Assessed by NICHD and Jensen Scoring Systems. The Eastern Society for Pediatric Research, Philadelphia, PA. (2024)” to “Cocuzzo, B & Kase, J. Prediction of Neurodevelopmental Impairment Based on Severity of Bronchopulmonary Dysplasia as Assessed by NICHD and Jensen Scoring Systems. The Eastern Society for Pediatric Research, Philadelphia, PA. (2024). (Abstract) “https://apps.eastern-spr.org/abstracts/?page=view&id=660”. The original article has been corrected.

